# Therapeutic Targeting of Minimal Residual Disease to Prevent Late Recurrence in Hormone-Receptor Positive Breast Cancer: Challenges and New Approaches

**DOI:** 10.3389/fonc.2021.667397

**Published:** 2022-02-10

**Authors:** David W. Cescon, Kevin Kalinsky, Heather A. Parsons, Karen Lisa Smith, Patricia A. Spears, Alexandra Thomas, Fengmin Zhao, Angela DeMichele

**Affiliations:** ^1^ Princess Margaret Cancer Centre, University Health Network, Toronto, CA, Canada; ^2^ Winship Cancer Institute at Emory University, Atlanta, GA, United States; ^3^ Department of Medical Oncology, Division of Breast Oncology, Dana Farber Cancer Institute, Boston, MA, United States; ^4^ Johns Hopkins University School of Medicine, Baltimore, MD, United States; ^5^ Lineberger Comprehensive Cancer Center, University of North Carolina, Chapel Hill, NC, United States; ^6^ Division of Hematology and Oncology, Wake Forest Baptist Comprehensive Cancer Center, Wake Forest University, Winston-Salem, NC, United States; ^7^ Dana Farber Cancer Institute – ECOG-ACRIN Biostatistics Center, Boston, MA, United States; ^8^ Abramson Cancer Center, University of Pennsylvania, Philadelphia, PA, United States

**Keywords:** tumor dormancy, minimal residual disease (MRD), ctDNA = circulating tumor DNA, molecular residual disease, CTC = circulating tumor cell, breast cancer, adjuvant therapy

## Abstract

While the majority of breast cancers are diagnosed at a curable stage, approximately 20% of women will experience recurrence at a distant site during their lifetime. These metastatic recurrences are incurable with current therapeutic approaches. Over the past decade, the biologic mechanisms underlying these recurrences have been elucidated, establishing the existence of minimal residual disease in the form of circulating micrometastases and dormant disease, primarily in the bone marrow. Numerous technologies are now available to detect minimal residual disease (MRD) after breast cancer treatment, but it is yet unknown how to best target and eradicate these cells, and whether clearance of detectable disease prior to the formation of overt metastases can prevent ultimate progression and death. Clinical trials to test this hypothesis are challenging due to the rare nature of MRD in the blood and bone marrow, resulting in the need to screen a large number of survivors to identify those for study. Use of prognostic molecular tools may be able to direct screening to those patients most likely to harbor MRD, but the relationship between these predictors and MRD detection is as yet undefined. Further challenges include the lack of a definitive assay for MRD with established clinical utility, difficulty in selecting potential interventions due to limitations in understanding the biology of MRD, and the emotional impact of detecting MRD in patients who have completed definitive treatment and have no evidence of overt metastatic disease. This review provides a roadmap for tackling these challenges in the design and implementation of interventional clinical trials aimed at eliminating MRD and ultimately preventing metastatic disease to improve survival from this disease, with a specific focus on late recurrences in ER+ breast cancer.

## Introduction

In 2019 in the United States, approximately 270,000 new cases of invasive breast cancer were diagnosed and approximately 42,000 women died of the disease. Over 90% of these cases were diagnosed in stages I – III, at a point at which they are potentially curable. Once the disease has left the breast and axillary lymph nodes and become clinically detectable in distant organs, it is no longer curable. Deaths from breast cancer are due to metastatic disease to distant sites that interfere with normal bodily functions. It is estimated that approximately 3.8 million women in the United States have been treated for stage I-III breast cancer. Unfortunately, up to 20% of these patients will experience recurrence at a distant site in their lifetime (1). There is a critical unmet need to identify which women are most likely to recur and to prevent recurrence before it can manifest as incurable overt metastatic disease. The recurrence pattern from natural history studies of hormone-receptor positive (HR+) breast cancer demonstrates that the highest risk of relapse occurs in the first two years of follow-up, followed by a near constant annual relapse rate over the course of a lifetime (ranging up to approximately 2% per year in the highest risk patients), such that approximately 50% of the risk of recurrence for an individual woman is in the period beyond 5 years from diagnosis (2). “Early” recurrence typically refers to those recurrence events that take place within the first 3-5 years, while “late” recurrence typically refers to those events taking place 5 or more years from diagnosis. Adjuvant endocrine therapy, when given for 5 years after initial diagnosis and treatment, has been shown to reduce recurrence risk and improve survival (3, 4). However, this early treatment has less impact on late recurrence risk. The mechanisms driving late recurrences are not clear: both acquired resistance of dormant cells to sustained endocrine therapy or, conversely, the release from dormancy enabled by discontinuation of endocrine therapy have both been implicated (5, 6).

Recurrences that occur beyond 5 years are only modestly reduced by extending the same adjuvant endocrine therapy, i.e., tamoxifen or aromatase inhibitor, for an additional time period (7). Since many women experience late recurrences and extended adjuvant endocrine therapy benefits only a small fraction of women with HR+ breast cancer, dual challenges exist: to find better ways to identify women who are at risk for late recurrence, and develop therapeutic strategies that will further reduce the risk of recurrence in these women. To address these needs, leaders from the National Clinical Trials Network (NCTN) breast cancer committees convened a Clinical Trials Planning Meeting (CTPM) in May 2019, which was followed by an extended planning process to identify new approaches to these issues. This review summarizes the goals and challenges of designing a trial for late recurrence in HR+ breast cancer and recommendations for future trials.

### Designing a Trial for Late Recurrence: Challenges and Opportunities

**Figure 1:**
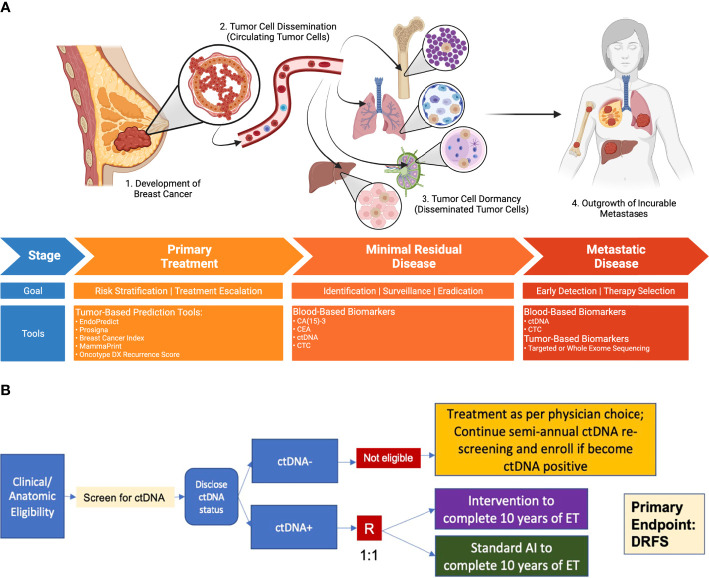
Process by which primary tumors progress through dormancy to distant metastatic disease, and opportunities for intervention. **(A)** Breast Cancer Treatment Continuum: Minimal Residual Disease as a Therapeutic Opportunity to Prevent Late Recurrence. New approaches to identify at-risk individuals and evaluate therapies to reduce late recurrence may focus on the period following standard upfront treatment. The detection of minimal residual disease through blood-based surveillance tools might enable the identification of individuals at highest risk of metastatic recurrence, for whom escalated therapies may have the greatest potential benefit. The principal goal of such interventions is the prevention of metastatic recurrence. **(B)** Example Design Schema for a Phase 3 ctDNA-Guided Late Recurrence Trial. The use of highly sensitive ctDNA detection methods in patients at high clinical risk permits the identification of those most likely to recur, for whom investigational therapies could be evaluated (bottom). Those without detectable ctDNA may continue regular ctDNA surveillance, becoming eligible for therapeutic intervention if ctDNA is subsequently detected. Clinical endpoints of particular importance include distant recurrence-free survival, and overall survival. “R” denotes randomization step; DRFS, distant recurrence free survival; ET, endocrine therapy.

Any trial designed to reduce the risk of late recurrence has several required elements for success, as outlined in **Table 1**. First, it is necessary to identify the population at risk for late recurrence. Several molecular tumor assays have been developed with this goal, and their limited success is described below. All of these are imperfect, as they identify a relatively large population of patients at risk, of whom only a fraction will ultimately relapse. To avoid overtreatment, the ideal approach would be to identify a “real time” biomarker that reflects the presence of residual disease emerging from dormancy where recurrence is imminent but has not yet occurred. Capturing patients at a time when they are in this modifiable window of opportunity allows for intervention to prevent recurrence. Next, a successful trial requires an intervention that is effective against the disease that would otherwise recur, either eliminating these cells or reverting them to a state of permanent dormancy. Such an intervention could be targeted to the cells, the microenvironment in which they emerge, or the immune system, heightening immunosurveillance. Finally, a trial targeting late recurrence must address the complex needs of patients in this setting, who have no signs of overt disease, including the psychological effects of identifying residual disease, the physical and financial toxicity of therapy and the implications of a successful strategy: the need to be monitored and screened over the course of a lifetime. These issues and the various strategies that could be employed are discussed in the sections that follow, with key elements summarized in **Figure 1**.

**Table 1:** Design components needed for a late recurrence trial.

Design Component	Description	Examples, Challenges
**Method to identify patients at risk**	Molecular Tumor Assay	eg. Gene-expression assays (see **Table 2**), other tumor genomic characteristics
	Minimal Residual Disease Assay	eg. Tumor informed (“bespoke”) or agnostic ctDNA assays; circulating tumor cells. *Sensitivity of various approaches are incompletely characterized. Relationship between assay positivity and standard radiographic imaging is unknown.*
**Intervention**	Pharmaceutical or other intervention demonstrated to reduce both the MRD biomarker and recurrence	eg. endocrine, targeted or immunotherapy. *Tolerability or toxicity may be limiting for many potential interventions*
**Endpoints**	Clinical endpoint upon which to base success of the intervention	eg. metastasis-free survival. *Most relevant to the goal of the trial but not accepted FDA endpoint for drug registration. Long follow-up may be required.*
**Patient Reported Outcomes (PRO)**	Instruments that assess impact of identifying MRD on quality of life	eg. Global QOL, CTCAE-PRO.

## Tumor Dormancy and Minimal Residual Disease Detection in Breast Cancer

The fact that HR-positive breast cancer can recur many years (or even decades) following diagnosis and treatment for primary disease suggests one of two possibilities: residual tumor cells disseminated in the body proliferate at a very slow rate, until these growing tumors become clinically evident; or residual tumor cells lie dormant for prolonged periods, until they escape and grow more quickly to form detectable tumors. Conclusive evidence to support either the former (indolency) or latter (dormancy) model is lacking, and it is likely each phenomenon contributes to some cases of metastatic recurrence. However, based on many factors, including observed rates of tumor growth in primary disease and their recurrences, temporal and spatial patterns of recurrence, and biologic evidence to support the existence of dormant states (reviewed in other accompanying manuscripts), dormancy is favoured as a key contributor to late recurrences of HR+ disease (8, 9).

In either case, dissemination of microscopic disease that is not removed by locoregional treatment (surgery and radiation) is a necessary prerequisite. Circulating tumor cells (CTCs) in the blood are implicated in early dissemination (10). The existence of, and ability to detect disseminated tumor cells (DTCs) in the bone marrow of patients diagnosed with breast cancer has been well-recognized and studied for several decades. DTC detection frequencies of ~25-30% at the time of early breast cancer diagnosis have been observed in large, pooled analyses, where DTC detection is associated with high-risk tumor features, such as tumor size, nodal positivity, and tumor grade (11). In multivariable analyses, the presence of DTCs is independently associated with the risk of recurrence (though limited data are available to specifically assess late recurrence of HR+ disease) (12, 13). Bone marrow DTCs in patients without clinically evident metastases are one example of “minimal residual disease” (broadly defined as persistent evidence of cancer that is not detectable with standard clinical or radiographic assessments, MRD), which may provide the seeds for subsequent recurrence. However, their direct role in the metastatic cascade is uncertain, and not all patients with bone marrow DTCs at diagnosis experience recurrence. Numerous potential explanations for this exist, including the elimination of residual disease with adjuvant therapy, long term control or elimination by the host immune system, or a lack of the necessary cell-intrinsic or extrinsic (eg. microenvironmental) conditions to permit tumor outgrowth, resulting in either elimination of disseminated cells or their persistent dormancy.

An ability to identify and assess MRD over time would permit both a better understanding of its natural history, and perhaps better enable accurate individualized risk assessment at time points remote from diagnosis that could guide clinical interventions. Unfortunately, the bone marrow biopsies necessary to assess DTCs are invasive and uncomfortable, limiting their clinical application. However, the rapid development of several technologies for liquid biopsy from circulating blood offer some novel approaches to this problem. Liquid biopsy to detect MRD has focussed on two main approaches: (1) the detection of intact tumor cells in the circulation (circulating tumor cells, CTCs), and (2) the detection of DNA released from tumor cells detected in the circulation as cell-free DNA (circulating tumor DNA, ctDNA) (14). The technologies underlying each of these approaches (recently reviewed elsewhere (14, 15)) depend on the identification of tumor specific features not normally present in blood samples. For CTCs, this includes immunophenotypic characteristics of circulating cells such as EpCAM (Epithelial Cell Adhesion Molecule) and cytokeratin (together with absence of leukocyte markers) that mark their tumor origin (10), whereas for ctDNA, the presence of somatic alterations (point mutations, copy number alterations) or methylation patterns can distinguish ctDNA from other cell free DNA (cfDNA) released by normal cells.

Several recent reports have identified strong associations between ctDNA or CTC detection and subsequent breast cancer recurrence, as described below (16–20). In general, detection of MRD using current liquid biopsy techniques has yielded lead times (ie. the time between sampling and clinical presentation with metastatic disease) of about 1-2 years. While it is possible, or perhaps likely, that these lead times may increase as assay detection sensitivities increase, several important gaps exist in our current understanding of MRD detection that would impact its clinical utility. Chief among these is the proportion of detectable cases that represent true MRD, which could not be simultaneously detected using other standard imaging techniques, as opposed to radiographically overt but clinically occult metastatic disease. This distinction is crucial, since true MRD may present an opportunity for cure, whereas established breast cancer metastases are understood to be generally incurable with currently available therapies. Prospective studies that incorporate serial imaging with concurrent sampling for liquid biopsy will be required to address this question. A second important and unanswered question is whether detectable MRD represents disease that remains dormant but at risk for later escape, or represents instead a later stage of cancer outgrowth following escape from dormancy. This distinction is important, as relevant therapeutic strategies may differ in each scenario (discussed further below). Additional clinical evaluation of evolving liquid biopsy technologies should provide some insight, as will monitoring of dynamic changes in MRD characteristics in response to proposed therapeutic interventions. However, it remains unclear whether changing the therapeutic approach at the time of MRD detection or whether changes in MRD after modifying treatment will correlate with improved clinical outcomes.

Despite the existing uncertainties noted above, several studies have been recently been launched to investigate the use of ctDNA MRD as a selection marker for interventional trials. These include studies of HR+ and HR- breast cancer, which principally focus on the early recurrence period. c-TRAK-TN (NCT03145961) evaluated immunotherapy in patients with triple negative breast cancer, DARE (NCT04567420) and LEADER (NCT03285412) are evaluating CDK4/6-inhibitors in ER+ disease, and ZEST (NCT04915755) is evaluating a PARP inhibitor in BRCA-related or triple negative breast cancer.

The elimination of MRD to prevent breast cancer late recurrence and achieve clinical cures will require therapeutic intervention with systemic treatments that will bring costs – both financial and in the form of treatment toxicity. Ensuring that costs are accompanied by the greatest likelihood of benefit could be achieved by the identification of individuals most likely to recur. While in other adjuvant settings this is generally achieved by using population-based tools for risk stratification, it is possible that MRD detection could ultimately binarize individual risk (ie. if patients with MRD at late timepoints invariably experience recurrence). However, the current costs and practical logistics of liquid biopsy approaches (combined with the overall low risk in unselected women with HR+ breast cancer) will nonetheless require tailoring any MRD-based intervention strategy to populations with some meaningful risk threshold. The application of various existing risk-stratification tools based on standard clinicopathologic variables could permit the development of such a strategy. In the remainder of this review, we discuss the key considerations for the development and evaluation of a therapeutic strategy to prevent late recurrences of HR+ breast cancer.

## Identifying the Population at Risk

### Tumor-Based Features

The overarching goal of risk stratification is to identify a population at high enough risk for metastatic recurrence in whom escalating treatment may be warranted. While late risk of recurrence can continue beyond 10 years, many population-based tools have only examined the 5-10 year window (1, 2). Risk thresholds could be set at varying levels to enrich a study population, based on the intensity of the intervention of interest, with 10% to 15% risk of distant recurrence from years to 5 to 10 resulting in feasible trial size (e.g. up to several thousand patients) with the potential for meaningful results in a definitive Phase III adjuvant trial. While higher risk level thresholds could limit feasibility and the rate of accrual since these patients are fewer in number, inclusion of patients with higher risk would enrich for events, enabling a larger absolute magnitude of benefit, and thus a higher benefit/risk ratio for a given intervention.

Both anatomic and biologic tools based on the excised primary tumor exist to assess recurrence risk. Standard clinicopathologic features, including anatomic stage (tumor size and lymph node involvement) and grade of the original tumor continue to provide information on risk of recurrence for at least 20 years from diagnosis, with risk of distant recurrence beyond year 5 ranging from 10% to 41% in the Early Breast Cancer Trialist Collaborative Group analysis of outcomes for almost 63,000 trial participants (1). Many patients in this initial work had been treated on older studies, and an updated analysis of 86,000 participants on 110 trials found that with contemporary therapy (patients diagnosed after 2000) the risk of recurrence beyond year 5 is approximately 25%. A tool based on standard clinicopathologic features, the Clinical Treatment Score Post 5 Years (CTS5) has recently been developed and described (21). This web-based, widely available calculator uses initial tumor size, grade, patient age and nodal status to classify patients as low (<5%), intermediate (5-10%) or high risk (>10%) for distant recurrence during years 6-10 following diagnosis. While simple, based on readily available information, and validated in post-menopausal women, this tool is based on limited data for extremes of tumor size or nodal status, and diminished validity has been observed among premenopausal women (22).

Gene expression classifiers have provided important molecular insights into breast cancer recurrence, and the expression of many genes – including those involved in proliferation and estrogen signaling – is correlated with risk of recurrence. The goal of genomic assays is to reliably define the risk of recurrence so that patients are appropriately treated with adjuvant systemic therapy. Several commercial assays have been developed and validated as prognostic tools and are used routinely to stratify patients for the delivery of adjuvant therapy (**Table 2**). These assays, which are performed on primary tumor tissue, include the immunohistochemical 4 (IHC4) protein test, 21-gene Recurrence Score (OncotypeDx), PAM50 intrinsic subtype (ProSigna ROR; risk of recurrence), 12-gene EndoPredict Score (EPClin), 70-gene signature (MammaPrint), and 2-component Breast Cancer Index (BCI; HOXB13:IL17BR). These genomic assays offer prognostic information on the anticipated natural history and risk of recurrence, having been analyzed in prospective-retrospective studies and, depending upon the test, prospectively validated in large, randomized trials, such as TAILORx (OncotypeDX) (34) and MINDACT (MammaPrint) (32), and RxPONDER (OncotypeDX) (35). In addition, genomic assays have the potential for predictive utility to help guide individual treatment decisions, such as omission of adjuvant chemotherapy. For instance, in the overall population with an intermediate recurrence score in TAILORx, defined as RS between 11 and 25, patients with node negative disease did not experience a significant clinical benefit with the addition of chemotherapy to endocrine therapy at 9 years (34). RxPONDER extended this finding to post-menopausal women with 1-3 involved lymph nodes, in that there was no subgroup of post-menopausal women with a recurrence score of < 26 who benefited from the addition of chemotherapy at 5 years (33). Similarly, in the MINDACT study, the MammaPrint assay was able to identify patients with high clinical risk but low genomic risk who had a relatively favorable prognosis in the absence of systemic chemotherapy (32).

**Table 2:** Genomic risk assessment tools.

Tool	Description	Level of Evidence
COMBINED GENOMIC/CLINICAL		
EndoPredict (23–25),	RNA based, 12-gene assay combined with tumor size and nodal status, developed in pre- and post-menopausal women treated with tamoxifen	- Validated ~2600 post-menopausal women in ABCSG6/8 and TransATAC
Prosigna ROR (26–28),	PAM50-based 46 gene-signature developed in pre- and post-menopausal women treated withoutany adjuvant systemic therapy. Includes tumor size	- Validated in ~2100 women in ABCSG 8 and TransATAC and in ~2500 Danish women cohort for 10-year risk of recurrence

GENOMIC		
Breast Cancer Index (29, 30),	Combines the 2-gene HOXB13:IL17BR ratio with the molecular grade index from five proliferation genes in a linear model; developed in post-menopausal patients with HR-positive, node negative breast cancer. The node positive assay includes tumor size	- Developed on blinded retrospective analysis of 588 Swedish women treated on tamoxifen trial
MammaPrint (31, 32),	70-gene RNA expression profile	- Level 1 evidence for addition of systemic chemotherapy to adjuvant anti-estrogen therapy
Recurrence Score (OncotypeDx) (33)	21-gene signature developed in HR-positive, N0 patients. RxPONDER demonstrated discrimination extends to post-menopausal women with disease involvement in 1-3 nodes	- Designed to predict benefit of addition of systemic chemotherapy to adjuvant anti-estrogen therapy; Level 1 evidence for this

While these assays each have demonstrated prognostic utility within the first five years from diagnosis, one study performed a direct comparison of various assays to evaluate their relative prognostic capabilities from years 0-10. In the TransATAC trial, tumors from 774 of the postmenopausal women with HR+/HER2- breast cancer were characterized with the following tests: OncotypeDx, BCI, Prosigna ROR, EPClin, IHC4, and CTS5 (36). For late (between 5-10 years) distant recurrence, BCI, ROR, and EPClin provided independent prognostic information for women with node-negative disease, as well as in a small population of patients with node-positive breast cancer. These three assays can also identify a subset of patients with anatomically low risk tumors who are at higher risk of recurrence, or who have anatomically higher risk tumors that have genomically lower risk disease. An analysis of EP using separate clinical trial cohorts (ABCSG-6/8) found that 22% of patients with node negative disease had tumors that were EPClin score high (with a predicted 10-year distant recurrence-free rate of 87.0% (95% CI 82.6%-91.7%) and that 30% of patients with node positive breast cancer had tumors which were EPClin score low (10-year distant recurrence-free rate of 95.6% (95% CI 92.2%-99.1%) (23). Similarly, BCI has reported on two cohorts of patients with low anatomical risk (T1N0) breast cancer in which 32% and 36% of tumors were classified as BCI high risk associated with reduced distant recurrence-free survival 86.7% and 89.6% at years 5-15 and 5-10 compared with 95.4% and 98.4% for patients with BCI low risk tumors in each cohort respectively (37). In 402 patients with node-positive (N1) breast cancer, ~20% were classified as BCI low risk, with a 15-year distant recurrence risk of 1.3% (30, 38–40).

Taken together, the available data support the use of both anatomic and tumor-based genomic classifiers to identify patients at increased risk of late recurrence for inclusion in a study of late recurrence interventions. Targeting a 10 to 15% risk of recurrence over years 5 to 10, such an approach would include anatomically high risk (e.g. Stage III disease) and reserve eligibility based on genomic high risk to those who are anatomically at lower risk. Ideally, all enrolled patients would have tumor-based molecular testing for subsequent correlation of genomic and anatomic risk, including in the higher stage cohorts for which limited information is currently available. Some important unknowns remain in the application of these tools to define a trial population, including the relationship between genomic classifiers and the prevalence of detectable MRD, as well as the impact of emerging therapies which may be incorporated into early adjuvant therapy of high-risk disease (e.g. cyclin-dependent kinase 4/6 inhibitors (41)) on their prognostic estimates. Furthermore, limitations of the existing risk classifiers include a paucity of validation in pre-menopausal women for several of the approaches, and uncertainty in whether any assays may be predictive of therapeutic benefit for various treatments that may be considered.

### Blood-Based Biomarkers

There is significant interest in the clinical development of minimally invasive tests, such as a blood-based biomarkers, that could help determine if an individual patient is or remains at high risk of recurrence. Ideally, a reliable marker would detect the presence (or likelihood) of minimal residual disease (MRD, as described above) to identify individuals at high risk of recurrence prior to radiographically detectable incurable metastatic disease (e.g., during a period of tumor dormancy). These patients may benefit from modification or escalation of therapeutic strategies to ultimately decrease the likelihood of developing metastatic breast cancer. This is an attractive approach, as it may detect the development of resistant disease in real-time, as opposed to basing prognostic risk upon clinical, pathologic or genomic features of the historical primary tumor.

While serial assessment of tumor markers, such as cancer antigen (CA)15-3 (the soluble moiety of the MUC-1 glycoprotein) and carcinoembryonic antigen (CEA) are used sometimes in patients with metastatic breast cancer, serum tumor markers are not recommended in the surveillance of patients treated for early breast cancer due to (i) concerns with sensitivity, (ii) the finding that the positive predictive values of these markers decrease over time, and (iii) lack of evidence that serum marker measurement improves clinical outcomes (42, 43). A renewed interest in the assessment of circulating markers has emerged with the development of newer tests for MRD, including CTCs and ctDNA, and emerging clinical results demonstrating their prognostic effects, including in the setting of late recurrence.

In the Eastern Cooperative Oncology Group E5103 trial, the presence of CTCs (i.e., at least 1 CTC per 7.5 cc whole blood) detected 4.5-7.5 years following diagnosis, as measured using the EpCAM-based CellSearch platform, was associated with an adjusted relative risk for distant recurrence of 13.1 [95% CI: 4.7 to 36.3] after a median follow-up of 2.6 years (41). Of note, only 5% of patients in E5103 were identified as having CTCs present. The median time to recurrence was 2.8 years (range, 0.1-2.8 years) among the CTC-positive patients. In a second study. (SUCCESS A), the presence of CTCs 5 years after chemotherapy in patients with HR+ breast cancer was also shown to be an independent predictor of recurrence-free survival in multivariable analysis (HR 5.95, 95% CI: 1.14 – 31.16, p = 0.035) (17). Studies with other platforms, including non-EpCAM based technologies, are ongoing to further evaluate the potential prognostic and predictive effects of CTCs. Recognizing that the studies described did not incorporate serial radiographic imaging (which is not routinely performed in this setting), the degree to which CTC detection in these studies represented true MRD is unknown.

In addition, a number of recent prospective-retrospective studies have described the evaluation of minimal residual disease (or in this case, “molecular residual disease”) with ctDNA in patients with early-stage breast cancer. Some platforms have used bespoke assays which target somatic alterations identified in the primary tumor (18) while others are agnostic to alterations identified in the primary (19). In a cohort of 144 patients and at a median follow-up of 36.3 months, molecular residual disease was detected in 29 patients, which was highly prognostic in a time-dependent model (HR, 32.8; 95% CI, 13.5-79.2; P < .001). The median lead time between ctDNA detection and relapse was 10.7 months (95% CI, 8.1-19.1 months) (18). While this analysis included all breast cancer subtypes, 51 patients had HR+/HER2- breast cancer, and the hazard ratio was not definable because no patients experienced relapse in the ctDNA-negative group, with a median lead time of 13.3 months for those with ctDNA-positivity (95% CI, 2.1 months to undefined; P < .001). In addition to identifying MRD and its associated risk of recurrence, ctDNA analysis can also identify genomic alterations, such as ESR1 mutations (which confer ligand-independent activation of the estrogen receptor), which may help define mechanisms of resistance to standard therapy (eg. aromatase inhibitors) and inform therapeutic interventions, as well as provide opportunities to assess response to these treatments, such as by dynamic changes in variant allele frequency (16).

## Incorporation of Markers Into a Prospective Therapeutic Trial

While CTCs and ctDNA have the potential to further discriminate risk, predict potential benefit to therapy or provide surrogate markers of response, these blood markers are not yet ready for inclusion in a definitive clinical trial in the late adjuvant setting. An integral marker for a large adjuvant study must satisfy a number of criteria, which - in the case of CTCs and ctDNA for late recurrence - remain to be further defined. Chief among these, from a screening perspective, include the relationship between minimal residual disease positivity *via* CTCs or ctDNA and scan-detectable, subclinical metastatic disease, and the prevalence of CTC or ctDNA positivity in a population eligible for a large adjuvant study. It also remains unclear whether the identification of these markers represents a tumor that has already escaped from dormancy or is at imminent risk of doing so. Further investigation will also clarify if the detection of CTCs and ctDNA capture similar or distinct groups of patients during the trajectory of recurrence, and thus whether they are interchangeable or complementary; for instance, are CTCs generally present before the appearance of ctDNA or vice versa? Additionally, relationships between blood-based markers and predicted risk based upon baseline tumor genomic signatures and dynamic changes in markers with the introduction of a new therapeutic strategy are not defined, and could inform an optimal trial strategy. While there are a number of pre-analytic and analytic considerations with these tests, the field is rapidly evolving, and we anticipate that there will be a role for liquid biopsy as prognostic and predictive biomarker, given the ability to easily collect and monitor these features over time.

## Potential Therapies for Late Recurrence

Therapeutic intervention to reduce the risk of late recurrence of ER-positive breast cancer will require agent(s) that are effective, safe and tolerable. Several classes of agents now available and under study in advanced breast cancer and in the earlier adjuvant setting could be considered in this setting.

Inhibition of cyclin-dependent kinases 4 and 6 (CDK4/6) in combination with hormonal therapy has been a highly effective treatment regimen for metastatic HR+ breast cancer with three FDA-approved agents – abemaciclib, palbociclib and ribociclib – showing similar outcomes with somewhat differing side effect profiles (44). Randomized studies of adjuvant CDK4/6 inhibitors in early breast cancer have recently reported contrasting results, with abemaciclib reducing the risk of recurrence at 1-2 years for patients with node-positive, high-risk, ER-positive breast cancer in the MONARCH-E trial (45), and palbociclib failing to show benefit in the PALLAS (46) and PENELOPE (47) trials. Further maturation of these data and the results from a similar study of ribociclib (the NATALEE trial) will provide a better understanding of the potential efficacy of these agents early in the course of adjuvant therapy for high risk disease. In addition, correlative studies are likely to provide additional insights to guide clinical implementation or additional study. While the mixed results of adjuvant trials reported to date are somewhat disappointing, CDK4/6 inhibitors remain of interest for the prevention of late recurrence, given their proven efficacy in the advanced setting, combined with their safety and tolerability in both advanced and early stage breast cancer.

Selective Estrogen Receptor Degrader/Downregulators (SERDs) have been highly effective in pre-clinical models, with substantial, but more limited efficacy in the clinical setting likely due to the difficulty in delivering an effective dose of the sole FDA-approved injectable SERD, fulvestrant. Fulvestrant is active in tamoxifen- and aromatase inhibitor-refractory cancers and is an established treatment either alone or in combination with targeted therapies for metastatic disease. A new class of orally-bioavailable SERDs shows promise in the advanced breast cancer setting both in terms of efficacy and tolerability, and would be particularly attractive as compared to a monthly injectable agent.

Additional agents that could be considered include selective inhibitors of the PI3K/AKT/mTOR pathway, which has a well-defined role in HR+ breast cancer. Currently available agents, however, are of more limited promise for application in the late recurrence setting. The FDA-approved PI3K inhibitor, alpelisib improves disease control but not survival in PIK3CA-mutant advanced HR+ disease when added to endocrine therapy. While the availability of a predictive biomarker (PIK3CA mutation) would permit genomically-guided adjuvant therapy, drug toxicities make it an unlikely candidate for the late adjuvant setting. AKT inhibitors, currently under evaluation in Phase III trials, are also promising agents for advanced breast cancer, but side effects are also likely to be limiting.

Beyond these agents that are approved or in advanced development for ER+ breast cancer, other strategies with relevance to the biology underlying tumor dormancy and escape could have potential relevance to future late recurrence prevention efforts. Given the likely contribution of anti-tumor immunity preventing metastatic recurrences, anti-cancer immunotherapy strategies such as immune checkpoint blockade could have obvious appeal. However, to date the understanding of the role of these therapies in ER+ breast cancer is limited and given their uncommon but potentially serious toxicity and high cost, much additional work to define at risk populations and potential predictors of benefit remains before late intervention trials could be considered. With continued investigation of the biology of tumor dormancy, innovative strategies may permit future approaches for drug repurposing or novel agent development in order to specifically address dormancy or reawakening (48). Any clinical investigation of such strategies will require careful assessment of drug safety and tolerability and attention to risk stratification and patient selection.

Timing and duration of any intervention to prevent late recurrence will also require examination in order to select an effective and tolerable regimen. Because the risk reduction due to five years of anti-estrogen therapy is well-established and substantial (49), intervening just after this period is attractive for both practical and biologic reasons. Most patients will continue through at least five years from diagnosis in the care of an oncologist, who could facilitate discussion and administration of additional treatment. Biologically, this time period may represent an increased release from dormancy at completion of the initial anti-estrogen treatment, and so an opportune time to intervene (9).

From numerous studies demonstrating that longer duration of adjuvant endocrine therapy improves outcomes, it follows that similarly cytostatic agents would require a significant duration of treatment for greatest efficacy. This must be balanced with acceptable tolerability and long-term safety for a group of patients who are otherwise clinically free from breast cancer and more than five years from initial diagnosis and treatment.

## Considering the Patient Perspective

When planning any interventional trial, one must consider issues related to the patient experience that may impact decisions to participate in the trial as well as the subsequent acceptability and uptake of the intervention if clinical benefit is demonstrated. To date, little is known about how patients make decisions regarding extended adjuvant therapy nor about their experience during this phase of treatment many years after initial diagnosis.

While is it well-established that HR+ breast cancer has an ongoing risk of recurrence that extends into the second decade after diagnosis, the manner in which patients perceive their risk of recurrence many years after diagnosis has not been rigorously studied. Additionally, the risk threshold beyond which patients may be willing to consider escalation of therapy after completion of 5 years of adjuvant endocrine therapy is not known. Data collected relatively soon after diagnosis indicate that patients with breast cancer often have inaccurate perceptions of their risk of recurrence, frequently over-estimating, but sometimes under-estimating, their risk (50–52).

In the early period following diagnosis, provider communication has been shown to impact accurate perception of risk (50, 53). Optimizing strategies to communicate the risk of late recurrence after completion of 5 years of adjuvant endocrine therapy will be critical for patients considering participating in a proposed trial escalating therapy after 5 years of initial endocrine therapy and, subsequently (should the intervention succeed), when implemented in routine clinical care. Communication about biomarker results has been shown to impact patients’ perception of their risk of late recurrence and subsequent decisions about extended adjuvant endocrine therapy  (54). Developing strategies for communicating the prognostic and predictive information provided by biomarkers – especially for novel and potentially strongly prognostic liquid biopsy markers of MRD – in the late adjuvant setting is a priority.

The potential psychological impacts associated with fear of recurrence that may accompany a determination of being at “high risk” of recurrence justifying treatment escalation must be considered. While the association between accurate perception of risk with anxiety, distress, depression and quality of life has not been rigorously studied, it is well established that breast cancer survivors frequently experience long-lasting adverse psychological outcomes and that fear of recurrence is associated with depression, anxiety and lower quality of life (50, 55–57). For the individual patient, learning that recurrence risk remains high after completing 5 years of adjuvant endocrine therapy (in addition to their earlier primary surgery, as well as any radiation and chemotherapy) may be an unwelcome surprise. While identifying an effective therapeutic option to reduce this risk is the overall goal of late recurrence elimination strategies, it is critical that the emotional impact associated with learning about residual risk and being offered escalation of therapy so many years after diagnosis is assessed.

The degree of risk reduction conferred by a potential intervention that patients would deem worthwhile in the late adjuvant setting must also be understood. Available data suggest that many patients are willing to consider extended adjuvant endocrine therapy even if the expected risk reduction is small (56, 58). In a survey of 112 patients with stage I-III breast cancer who had completed 3-5 years of adjuvant endocrine therapy, 52% indicated they were at least “moderately” willing to consider extended endocrine therapy for only a 1% absolute reduction in the risk of recurrence, while 78% and 89% were similarly willing for expected absolute benefits of 5% and 20% respectively.

Knowledge of patient willingness to extend therapy is based on continuation of endocrine therapy with which they have personal experience, or the escalation of therapy involving addition or switching to alternative endocrine agents. In this patient population, toxicity will be an important factor when deciding to add a new treatment with different toxicities, balancing baseline risk and expected risk reduction with toxicities. Side effects such as hot flashes, sexual problems and musculoskeletal discomfort are common among patients receiving adjuvant endocrine therapy (59). The toxicity profile associated with CDK4/6 inhibitors administered in conjunction with endocrine therapy in the metastatic and adjuvant settings is generally favorable (60), though may be perceived differently in the adjuvant setting, where rates of treatment discontinuation observed in the recently reported early adjuvant trials are significant. Assessment of treatment toxicity using patient-reported outcome measures will be a key component of evaluation of a proposed treatment escalation strategy, as patient tolerance may differ in the late adjuvant setting than in the settings in which these agents have previously been evaluated. Additionally, expanding our understanding of the toxicity associated with other potential therapies, including oral SERDs discussed above, will be an important component of ongoing research.

Even very promising interventions will not successfully reduce the risk of late recurrence of HR+ breast cancer if patients do not actually take them. Not only must willingness to pursue the therapy be considered, but also adherence during therapy (taking the intervention regularly as prescribed) and persistence during therapy (not discontinuing prior to completion of the planned course). Up to approximately 50% of patients have poor adherence to the first 5 years of adjuvant endocrine therapy or discontinue adjuvant endocrine therapy prior to completing 5 years (61). Multiple factors, such as age, beliefs about medication necessity, co-payment, side effects and more are associated with poor adherence and early discontinuation of adjuvant endocrine therapy (62–65). In trials evaluating extended adjuvant endocrine therapy, approximately 40% of participants discontinued therapy early (66). Premature treatment discontinuation in the phase III trials evaluating palbociclib and abemaciclib in the early adjuvant setting occurred in 42% and 27% of participants (45, 46), with the majority of early discontinuation due to toxicity. However, it is not known whether these findings can be extrapolated to the late adjuvant setting as tolerance may differ. Factors associated with adherence and persistence with late escalation of therapy by adding a CDK4/6 inhibitor and/or SERD must be defined. Patient tolerance and reasons for nonadherence or discontinuation can only be determined using appropriate patient reported outcome measurements. Examination of adherence as well as symptoms and side effects, quality of life, fear of recurrence, anxiety and risk perception must be assessed at regular intervals during interventional trials to determine how treatment influences adherence. These endpoints are important adjuncts to efficacy data that will inform whether escalation of therapy after 5 years with one or both of these interventions delivers clinical benefit in this setting. If late adjuvant therapies demonstrate efficacy and escalated late adjuvant therapy becomes a standard of care for patients at high risk for late recurrence, future research will need to identify potential barriers to uptake, adherence and persistence in the real-world setting.

## Summary

In summary, patients with a history of HR+ early breast cancer have a sustained, ongoing risk of metastatic recurrence over the course of their lifetime. Standard use of adjuvant endocrine therapy for an initial five years has modulated that risk, but it continues to persist, even with the advent of extended adjuvant therapy from years 5 - 10. New strategies are needed to reduce or eliminate this risk in order to reduce breast cancer mortality. Challenges of clinical trials in this setting include identifying patients who are ultimately destined to recur to avoid overtreatment, and identification of effective agents to successfully target the biology of potentially dormant minimal residual disease. The recent development of technologies such as tumor genomic assays that complement clinicopathologic risk assessment and blood-based assays, including CTCs and ctDNA to identify patients harboring MRD, provide an opportunity to test new therapies in this setting in patients at greatest risk. Available agents such as CDK4/6 inhibitors and oral SERDs, which have demonstrable clinical activity in advanced breast cancer, may overcome resistance to standard endocrine therapy that enables dormant tumors to emerge and spread. While these individual components provide promising tools to enable the development of late recurrence strategies, designing trials to take advantage of these advances remains challenging due to numerous unanswered questions. The prevalence of blood-based tumor markers, and their relationship with radiographically detectable disease in patients years out from treatment must be understood for the statistical design of such trials. The optimal assay(s) to measure and track MRD remain uncertain, as do the risk attributable to ctDNA and/or CTCs and lead time prior to overt metastatic disease. Most importantly, additional data to determine whether eradicating ctDNA and/or CTCs will result in reduction in recurrence and improved survival such that these assays could serve as surrogate measures is lacking. Finally, impacts on patients, ranging from the emotional impacts of diagnostic risk information to the toxicities of long-term therapy, and their resulting effects on quality of life and on treatment adherence and persistence are areas of significant uncertainty. The optimal approach to studying new agents in this setting will require incorporating patient reported outcomes assessment to understand these issues and successfully test new agents and strategies to ultimately eliminate late recurrence and death.

We have developed a collaboration spanning NCTN groups “REFINE-BrCa” (REFining Adjuvant Therapy through Identification aNd Escalation) to develop clinical trials and address existing critical knowledge gaps. An initial Phase 2 study is planned to evaluate co-primary endpoints of treatment persistence and MRD clearance in patients with high-risk ER+/HER2-negative disease upon switch to oral SERD after 4-8 years of endocrine therapy. This study will provide key data on the performance of ctDNA assays for detection and response evaluation in the extended endocrine setting. We anticipate the results of this study will rapidly inform clinical development in this field and enable the launch of a definitive Phase 3 trial (**Figure 1B**).

## Author Contributions

AD, DC, KK, KS, AT, HP, FZ, and PS contributed to the conceptualization and writing of the manuscript, contributing individual components and editing the whole. All authors contributed to the article and approved the submitted version.

## Funding

Funds for open access publication fees were provided by the Abramson Cancer Center.

## Conflict of Interest

DWC, consulting/advisory role for Agendia, AstraZeneca, Dynamo Therapeutics, Eisai, Exact Sciences, GlaxoSmithKline, Merck, Pfizer, Puma Biotechnology, Gilead, and Roche; research support (to institution) from GlaxoSmithKline, Inivata, Merck, Pfizer and Roche outside of the submitted work; patent for methods of treating cancers characterized by a high expression level of spindle and kinetochore associated complex subunit 3(ska3) gene (US62/675,228). KK, consulting/advisory role for Eli-Lilly, Pfizer, Novartis, Eisai, AstraZeneca, Immunomedics, Merck, SeattleGenetics, and Cyclocel; stock ownership (spouse) in Grail, Array BioPharma; former employment relationship (spouse) with Pfizer. HAP, research support (to institution) from Puma Biotechnology outside of the submitted work. KS, research support (to institution) from Pfizer outside of the submitted work; stock ownership (spouse) in ABT Labs and Abbvie. PAS, advisory committee role for Pfizer outside of the submitted work. AT, advisory role for BeyondSpring Pharmaceuticals; research funding (to institution) from Sanofi; stock ownership in Gilead Sciences, Bristol-Myers Squibb and Pfizer, and stock ownership (spouse) in Johnson and Johnson; royalties from Up-To-Date (spouse). AD research support (to institution) from Novartis, Pfizer, Genentech, Calithera outside of the submitted work.

The remaining author declares that the research was conducted in the absence of any commercial or financial relationships that could be construed as a potential conflict of interest.

## Publisher’s Note

All claims expressed in this article are solely those of the authors and do not necessarily represent those of their affiliated organizations, or those of the publisher, the editors and the reviewers. Any product that may be evaluated in this article, or claim that may be made by its manufacturer, is not guaranteed or endorsed by the publisher.
